# Effects of acid-base variables and the role of carbonic anhydrase on oxalate secretion by the mouse intestine in vitro

**DOI:** 10.14814/phy2.12282

**Published:** 2015-02-25

**Authors:** Jonathan M Whittamore, Susan C Frost, Marguerite Hatch

**Affiliations:** 1 Department of Pathology, Immunology and Laboratory Medicine, College of Medicine, University of FloridaGainesville, Florida, USA; 2 Department of Biochemistry and Molecular Biology, College of Medicine, University of FloridaGainesville, Florida, USA

**Keywords:** Anion exchange, bicarbonate, chloride, CO _2_, colon, ileum, pH

## Abstract

Hyperoxaluria is a major risk factor for calcium oxalate kidney stones and the intestine is recognized as an important extra-renal pathway for eliminating oxalate. The membrane-bound chloride/bicarbonate (Cl^−^/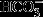) exchangers are involved in the transcellular movement of oxalate, but little is understood about how they might be regulated. 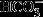, CO_2_, and pH are established modulators of intestinal NaCl cotransport, involving Na^+^/H^+^ and Cl^−^/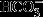 exchange, but their influence on oxalate transport is unknown. Measuring ^14^C-oxalate and ^36^Cl fluxes across isolated, short-circuited segments of the mouse distal ileum and distal colon we examined the role of these acid-base variables and carbonic anhydrase (CA) in oxalate and Cl^−^ transport. In standard buffer both segments performed net oxalate secretion (and Cl^−^ absorption), but only the colon, and the secretory 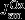 pathway were responsive to 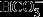 and CO_2_. Ethoxzolamide abolished net oxalate secretion by the distal colon, and when used in tandem with an impermeant CA inhibitor, signaled an intracellular CA isozyme was required for secretion. There was a clear dependence on 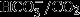 as their removal eliminated secretion, while at 42 mmol/L 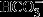 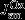 was also decreased and 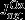 eradicated. Independent of pH, raising Pco_2_ from 28 to 64 mmHg acutely stimulated net oxalate secretion 41%. In summary, oxalate secretion by the distal colon was dependent on 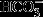, CA and specifically modulated by CO_2_, whereas the ileum was remarkably unresponsive. These findings highlight the distinct segmental heterogeneity along the intestine, providing new insights into the oxalate transport mechanism and how it might be regulated.

## Introduction

The kidney is the primary route for eliminating the metabolite oxalate from the body, but the intestine also possesses an important role in systemic oxalate homeostasis. Aside from absorbing dietary oxalate, the intestine has the capacity for oxalate secretion which is recognized as a valuable extra-renal pathway for its elimination (Hatch and Freel 2005, 2008; Robijn et al. 2011). Prior studies with isolated epithelial sheets, membrane vesicles, and cultured cell monolayers have all demonstrated that oxalate transport by the mammalian intestine is mediated, in large part, by an anion exchange mechanism (Hatch and Freel 2005, 2008). The anion exchangers expressed in the intestine primarily function as chloride/bicarbonate (Cl^−^/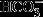) exchangers and molecular advances have identified individual anion transporters belonging to two distinct gene families, Slc4 and Slc26. In terms of transporting oxalate, the latter group has received the most attention, and development of knock-out (KO) mouse models for some members of this multifunctional Slc26 family have permitted their respective contributions to intestinal oxalate transport and overall homeostasis to be defined.

In the small intestine, PAT1 (Putative Anion Transporter 1; Slc26a6) is a Cl^−^/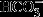 exchanger expressed predominantly in the small intestine, located in the apical membrane of the villus epithelium, with roles in nutrient and nonnutrient-linked Cl^−^ absorption, as well as 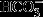 secretion and absorption, and intracellular pH regulation (Wang et al. 2002, 2005; Simpson et al. 2007, 2010; Singh et al. 2008, 2010; Walker et al. 2011; Xia et al. 2014). Studies of PAT1-KO mice have shown this transporter is also involved in oxalate secretion by the duodenum (Jiang et al. 2006) and ileum (Freel et al. 2006). Another important apical Cl^−^/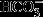 exchanger is DRA (Down-Regulated in Adenoma; Slc26a3), which is prominently involved in Cl^−^ absorption and 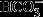 secretion along the length of the intestine (Walker et al. 2008; Singh et al. 2010; Freel et al. 2013; Whittamore et al. 2013; Xia et al. 2014; Xiao et al. 2014). Most recently, using DRA-KO mice we have shown that DRA also contributes to oxalate absorption by the ileum, cecum, and distal colon (Freel et al. 2013). The development of transgenic mice for these transporters has revealed the tremendous value of such animal models for advancing our understanding of intestinal oxalate transport and the roles of individual anion exchangers. Yet, there remains scant information on some of the overall fundamental oxalate-transporting characteristics of the mouse intestine, and furthermore how they might be regulated. Such knowledge will aid a better understanding of the patho-physiology behind disorders of oxalate metabolism, and is vital given the interest in developing the therapeutic potential of the intestine as a means to combat hyperoxaluria (Hatch et al. 2011; Robijn et al. 2011).

One of the most essential functions of the apical Cl^−^/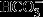 exchangers in the intestine occurs when coupled with sodium/proton (Na^+^/H^+^) exchange to perform electroneutral NaCl and fluid absorption. This is a highly regulated process and a broad range of hormonal, immune, and nervous system inputs can modify and coordinate NaCl cotransport and 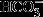 secretion by the intestine in a segment-specific manner (Kato and Romero 2011). The effects on Na^+^/H^+^ and Cl^−^/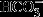 exchange activity in the ileum and colon in response to alterations in systemic acid-base homeostasis has previously been established for humans, rats, and rabbits in vivo and in vitro (Charney and Feldman 1984; Charney et al. 1995; Charney and Dagher 1996; Gennari and Weise 2008), and subsequently for mice in vitro (Goldfarb et al. 2000; Charney et al. 2004b). Elegant studies on the acute effects of CO_2_ in the rat distal colon demonstrated rapid shifts in the expression of these transporters at the apical membrane signaled through intracellular pH and [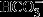], mediated by the catalytic enzyme carbonic anhydrase (CA) (Charney et al. 2002b, 2004a). The components of the bicarbonate buffering system (pH, 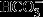 and CO_2_) therefore represent additional, and often overlooked, regulatory factors controlling intestinal electrolyte transport (Goldfarb et al. 1988; Charney and Dagher 1996), but their effects on oxalate handling have not been considered. As oxalate is a substrate of the Cl^−^/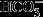 exchangers, we hypothesized that it too would display characteristics of being modified and regulated by these acid-base variables. The aim of this study was therefore to systematically examine how changes to pH, 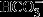 and CO_2_ impact oxalate transport by the mouse intestine in vitro. Given we know relatively more about intestinal Cl^−^ transport than oxalate, and because Cl^−^ is one of the primary substrates of the anion exchangers, collecting information on the associated Cl^−^ fluxes as part of these investigations offered a useful reference for interpreting the responses of oxalate in the context of what we presently understand about Cl^−^ transport. The following study reports how oxalate and Cl^−^ fluxes across the mouse distal ileum and distal colon were affected by alterations in acid-base variables and the role of CA.

## Materials and Methods

### Experimental animals

The following experiments used the wild-type (WT) C57BL/6 mouse strain. All mice were from breeding colonies housed at the AAALAC (Association for Assessment and Accreditation of Laboratory Animal Care)-accredited animal facility within the Biomedical Sciences Building at the University of Florida, where they were given free access to standard mouse chow (diet 2018S; Harlan Teklad) and water. For flux studies, male and female mice aged 2–5 months (20–30 g body mass) were killed by cervical dislocation following prior sedation induced by brief inhalation of isoflurane (≤15 sec). The entire lower portion of the intestinal tract (proximal ileum to distal colon) was then dissected out and placed in ice-cold buffer for immediate preparation for transport experiments. All animal experimentation was approved by the University of Florida Institutional Animal Care and Use Committee (IACUC) and performed in accordance with the National Institutes of Health “Guide for the Care and Use of Laboratory Animals.”

### Epithelial transport experiments

Unidirectional fluxes of oxalate and Cl^−^ were measured simultaneously under symmetrical, short-circuit conditions across pairs of intact, isolated tissues taken from the distal ileum (4 cm length immediately proximal to the ileo-cecal valve) and distal colon (4 cm length immediately proximal to the peritoneal border and representing the lower 30% of the large intestine). After removing the outer connective tissue each segment was opened longitudinally along the mesenteric border to form a flat sheet. From each segment a pair of tissues were prepared with each one mounted on a slider (P2304; Physiologic Instruments, San Diego, CA), exposing a gross surface area of 0.3 cm^2^, and secured into a modified Ussing chamber (P2300). The mucosal and serosal surfaces were bathed with 4 mL buffered saline and maintained at 37°C while being simultaneously gassed and stirred with the appropriate hydrated gas mixture (Table1). Each individual preparation was continuously voltage clamped to 0 mV with an automatic voltage clamp (model VCCMC6, Physiologic Instruments, San Diego, CA).

**Table 1:** The nominal concentration (mmol/L) of salts composing the buffers used in the following study. Each bicarbonate-containing buffer was equilibrated with a 95% O_2_/5% CO_2_ gas mixture at 37°C prior to measuring pH, total CO_2_, and osmolality. To elevate Pco_2_ in the standard, 21 mmol/L 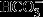 buffer an 89% O_2_/11% CO_2_ mixture was used.

Salt	Bicarbonate				Bicarbonate-free		
	7 mmol/L	21 mmol/L	21 mmol/L (High CO_2_)	42 mmol/L	pH 6.9	pH 7.4	pH 7.9
NaCl	118.4	118.4	118.4	97.4	118.4	118.4	118.4
K_2_HPO4	2.4	2.4	2.4	2.4	2.4	2.4	2.4
KH_2_PO4	0.6	0.6	0.6	0.6	0.6	0.6	0.6
NaHCO_3_	7.0	21.0	21.0	42.0	–	–	–
MgSO_4_	0.5	0.5	0.5	0.5	0.5	0.5	0.5
CaCl_2_	1.2	1.2	1.2	1.2	1.2	1.2	1.2
MgCl_2_	0.7	0.7	0.7	0.7	0.7	0.7	0.7
HEPES (free acid)	–	–	–	–	21.0	14.0	7.0
HEPES (Na^+^-salt)	–	–	–	–	–	7.0	14.0
Na^+^-gluconate	14	–	–	–	–	–	–
Mannitol	–	–	–	–	13	8	–
Gas mixture	95% O_2_/5% CO_2_	95% O_2_/5% CO_2_	89% O_2_/11% CO_2_	95% O_2_/5% CO_2_	100% O_2_	100% O_2_	100% O_2_
pH at 37°C	6.993	7.460	7.135	7.745	6.761	7.261	7.736
Pco_2_ at 37°C (mmHg)	29.6	28.0	64.0	30.6	–	–	–
Osmolality (mOsm/kg)	276	272	281	278	269	270	269

To measure the transepithelial fluxes of oxalate and Cl^−^, 0.27 *μ*Ci of ^14^C-oxalate (specific activity 115 mCi/mmol), and 0.09 *μ*Ci of ^36^Cl (specific activity 571 *μ*Ci/mmol) were added to either the mucosal or serosal chamber and which was then designated as the “hot side”. The addition of ^14^C-oxalate to the “hot side” required the respective addition of 0.9 *μ*mol/l “cold” oxalate in the form of Na_2_Ox to achieve a final concentration of 1.5 *μ*mol/l oxalate. This was matched by 1.5 *μ*mol/l Na_2_Ox on the opposing “cold side”. At 15 min intervals 1 mL samples were taken from the “cold side” to detect the appearance of these tracers, along with a recording of short-circuit current (*μ*A) and open-circuit potential difference (mV). Each sample taken from the “cold side” was immediately replaced with 1 mL of warmed buffer. At the beginning and end of each experiment a 50 *μ*L sample was taken from the “hot side” and used to calculate the specific activity (dpm/mmol) of each isotope. The activity of ^14^C-oxalate and ^36^Cl in all samples was determined by liquid scintillation spectrophotometry (Beckman LS6500, Beckman-Coulter, Fullerton, CA) with quench correction following dissolution in 5 mL scintillation cocktail (Ecoscint A, National Diagnostics, Atlanta, GA). Using a series of external standards for each isotope, the validity of counting dual-labeled samples was independently established thus allowing the individual activities of ^14^C-oxalate and ^36^Cl to be calculated on the basis of their relative counting efficiencies after modifying the detection channels to minimize overlap in their respective energy spectra.

The epithelial responses to the CA inhibitors, ethoxzolamide, and N-3500, as well as to increased CO_2_ partial pressure (Pco_2_) were performed as part of a paired experimental design. This involved commencement of an initial “control” period (0–45 min; Period I), after which the designated treatment was applied and the effects recorded for a further 60 min (45–105 min; Period II). In a bid to try and distinguish the involvement of intracellular CA isoforms (CAI and CAII) from the extracellular apical membrane-bound CAIV (Goldfarb et al. 2000), we utilized the impermeant CA inhibitor N-3500 (Delacruz et al. 2010). To target the external CAIV, N-3500 was added to the mucosal bath at the end of the initial control period (Period I). At the conclusion of Period II the membrane-permeant ethoxzolamide was then applied for a final third period (105–165 min; Period III) to inhibit all CA activity. The response to ethoxzolamide was tested in all buffers, whereas the effects of increasing Pco_2_, and the impact of N-3500 (coupled with ethoxzolamide) were examined in standard 21 mmol/L 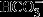 buffer only. For each buffer shown in Table1, the data collected during the initial control period of these paired experiments was subsequently used to independently compare the effects of varying [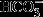] and pH.

### Buffer solutions and reagents

Table1 displays the nominal salt composition and characteristics of each buffer. The standard bicarbonate buffer contained 21 mmol/L 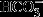 and was gassed with 95% O_2_/5% CO_2_ to achieve a pH and Pco_2_ of 7.4 and 28 mmHg, respectively. To reduce [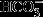] to 7 mmol/L, two-thirds of the NaHCO_3_ was replaced with 14 mmol/L sodium gluconate, and to help limit the increase in osmolality when increasing [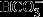] to 42 mmol/L, the [NaCl] was reduced by 21 mmol/L. The [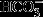] and Pco_2_ of each bicarbonate buffer was calculated following re-arrangement of the Henderson–Hasselbalch equation using measurements of pH and total CO_2_ (tCO_2_). pH was measured with an accupHast combination microelectrode (Fisher Scientific) connected to a Beckman 690 pH meter (Beckman-Coulter, Fullerton, CA), and tCO_2_ by a Corning 965 CO_2_ analyzer (Corning Ltd., Halstead, Essex, U.K.). To achieve a 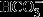/CO_2_ free buffer NaHCO_3_ was replaced with equimolar HEPES buffers and gassed with 100% O_2_. To modify pH under these conditions the concentrations of these complementary HEPES buffers were modified accordingly and in some cases necessitated the addition of mannitol to preserve osmolality (Table1). As part of this study was examining the effects of CA inhibition, the 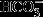/CO_2_-free buffers did not include a CA inhibitor. In all cases, the serosal buffer contained 10 mmol/L glucose, with an equivalent 10 mmol/L mannitol included in each mucosal buffer. To inhibit endogenous prostanoid production all buffers contained 5 *μ*mol/L indomethacin (Sigma, St. Louis, MO). The CA inhibitor ethoxzolamide was sourced from Sigma, and the 11% CO_2_/89% O_2_ gas mixture from Airgas Inc. A concentrated stock solution of ethoxzolamide in DMSO was made fresh on the day of each experiment and added to both mucosal and serosal chambers to a final concentration 100 *μ*mol/L, the resulting amount of DMSO in each half chamber was 0.05%. The impermeant CA inhibitor N-3500 was custom synthesized (Delacruz et al. 2010) and dissolved in standard 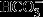 buffer prior to addition to the mucosal bath at a final concentration of 100 *μ*mol/L. The isotope ^14^C-oxalate was a custom preparation from ViTrax Radiochemicals (Placentia, CA) and ^36^Cl was purchased as HCl from Amersham Biosciences (Piscataway, NJ).

### Calculations and statistical analyses

The [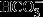] and Pco_2_ of each 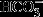/CO_2_-containing buffer was calculated from the measurements of pH and tCO_2_ (mmol/L) using the following re-arrangement of the Henderson–Hasselbalch equation: [dCO_2_] = [tCO_2_]/(1 + 10^(pH−pK″(aq))), where dCO_2_ is dissolved CO_2_, (mmol/L), and pK″(aq) is the first dissociation constant for carbonic acid corrected for the ionic strength and pH of the aqueous buffer solution (Siggaard-Andersen 1974). For the standard bicarbonate buffer at 37°C pK″(aq) was 6.118. The [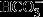] (mmol/L) was subsequently calculated by: [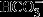] = [tCO_2_] − [dCO_2_], and Pco_2_ (mmHg) by: Pco_2_ = [dCO_2_]/*α*, where *α* is the solubility coefficient of CO_2_ (0.032 mmol/L•mmHg), corrected for the ionic strength of the buffer (Siggaard-Andersen 1974).

The fluxes of oxalate and Cl^−^ in the absorptive, mucosal to serosal (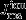) and secretory, serosal to mucosal (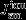) directions were calculated from the change in activity of ^14^C-oxalate or ^36^Cl detected on the “cold side” of the chamber at each 15 min sampling point, having corrected for dilution with replacement buffer between samples. These flux rates were expressed per cm^2^ of tissue surface area per hour. The recordings of short-circuit current (I_sc_; *μ*A) and transepithelial potential difference (mV) were used to calculate transepithelial conductance (G_t_; mS/cm^2^) following Ohm's Law. For each segment net fluxes (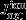) were calculated as: 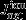 = 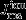 − 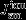 from tissues matched on the basis of conductance (no greater than a ± 15% difference in G_t_ between pairs of tissues from the distal ileum, and not exceeding ± 25% for tissue pairs from the distal colon).

The following data are presented as mean ± SE. For experiments conducted as a paired design, a repeated-measures, one-way ANOVA was used to evaluate the epithelial response to treatment with ethoxzolamide, 11% CO_2_, or N-3500 followed by ethoxzolamide, at each subsequent 15 min time point compared to the preceding control period. This control period value was taken as the mean of data points collected between 0 and 45 min. Significant differences following the experimental treatment were subsequently distinguished by multiple comparisons to the corresponding control value using Holm-Sidak post-hoc tests. Differences in flux rates and electrical characteristics in the presence of buffers with varying [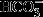] or pH were assessed by one-way ANOVA followed by Holm–Sidak multiple pairwise comparisons. For data failing to meet the assumptions of approximate normality and equality of variance, the equivalent nonparametric tests were performed. The results of all statistical tests were accepted as significant at *P* < 0.05. Statistical analysis was performed with SigmaStat v3.5 and the figures drawn using SigmaPlot v11.0 (Systat Software Inc. San Jose, CA).

## Results

### Effects of varying [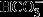]

Tables2 and 3 show that in standard buffer (21 mmol/L 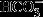) the distal ileum and distal colon mediated net oxalate secretion. In Table2, reducing extracellular [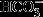] to 7 mmol/L or eliminating it altogether, did not significantly impact oxalate fluxes by the distal ileum. The latter condition, did result in net Cl^−^ secretion (−2.49 ± 1.24 *μ*mol/cm^2^/h), through a 30% decrease in 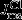. This indicates that even with CA activity intact, endogenous metabolic production of 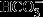 was insufficient to sustain Cl^−^ absorption and was thus dependent on an extracellular supply of 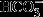/CO_2_. In this segment of the lower small intestine, when [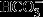] was increased to 42 mmol/L both oxalate and Cl^−^ fluxes were significantly affected cutting 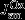 and 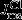 by 45 and 24%, respectively, with no accompanying alterations to *I*_sc_ or G_t_ (Table2). Conversely, in the distal colon (Table3), eliminating 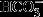/CO_2_ exclusively reduced 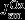 by 28% consequently abolishing net oxalate secretion by this segment. The net secretion of oxalate was also greatly diminished at 42 mmol/L 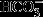, again through a decreased 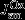 flux. Surprisingly, net Cl^−^ absorption was independent of [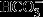], even in HEPES buffer where 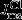 was not diminished, although 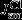 was 25% higher (Table3).

**Table 2:** Effects of [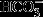] on oxalate and chloride transport by the distal ileum. A comparison of transepithelial oxalate (*J*^Ox^) and Cl^−^ (*J*^Cl^) fluxes measured simultaneously during period I (0–45 min) in buffers containing different concentrations of 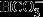 across pairs of isolated, short-circuited segments of the distal ileum from wild-type mice. Short-circuit current (*I*_sc_) and transepithelial conductance (*G*_t_) are also shown. Data are mean ± SE and values labeled with a different letter indicate a statistically significant difference. Numbers in parentheses denote sample size.

[ 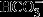 ] (mmol/L)	*J*^Ox^ (pmol/cm^2^/h)			*J*^Cl^ (*μ*mol/cm^2^/h)			*I*_sc_ (*μ*eq/cm^2^/h)	*G*_t_ (mS/cm^2^)
	*J*_ms_	*J*_sm_	*J*_net_	*J*_ms_	*J*_sm_	*J*_net_		
0 (pH 7.4)	16.70 ± 2.17 (8)	52.39 ± 5.50^a^ (8)	−35.69 ± 6.78 (8)	7.80 ± 0.65^a^ (8)	10.28 ± 1.15 (8)	−2.49 ± 1.24^a^ (8)	−1.95 ± 0.26 (16)	25.84 ± 1.81 (16)
7 (pH 6.9)	22.36 ± 5.74 (5)	56.35 ± 7.70^a^ (5)	−33.99 ± 5.64 (5)	11.01 ± 1.25^a,b^ (5)	10.19 ± 1.49 (5)	0.82 ± 1.23^a,b^ (5)	−3.37 ± 0.61 (10)	32.40 ± 3.90 (10)
21 (pH 7.4)	27.39 ± 3.69 (24)	52.26 ± 3.24^a^ (24)	−24.87 ± 4.78 (24)	11.16 ± 0.49^b^ (24)	9.43 ± 0.45 (24)	1.72 ± 0.62^b^ (24)	−2.49 ± 0.17 (56)	29.05 ± 0.98 (56)
42 (pH 7.9)	19.81 ± 3.55 (5)	28.99 ± 3.77^b^ (5)	−9.18 ± 5.70 (5)	8.45 ± 0.42^a,b^ (5)	8.49 ± 1.00 (5)	−0.04 ± 1.13^a,b^ (5)	−2.62 ± 0.36 (10)	31.43 ± 2.03 (10)

**Table 3:** Effects of [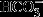] on oxalate and chloride transport by the distal colon. A comparison of transepithelial oxalate (*J*^Ox^) and Cl^−^ (*J*^Cl^) fluxes measured simultaneously during period I (0–45 min) in buffers containing different concentrations of 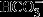 across pairs of isolated, short-circuited segments of the distal colon from wild-type mice. Short-circuit current (*I*_sc_) and transepithelial conductance (*G*_t_) are also shown. Data are mean ± SE and values labeled with a different letter indicate a statistically significant difference. Numbers in parentheses denote associated sample size.

[ 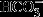 ] (mmol/L)	*J*^Ox^ (pmol/cm^2^/h)			*J*^Cl^ (*μ*mol/cm^2^/h)			*I*_sc_ (*μ*eq/cm^2^/h)	*G*_t_ (mS/cm^2^)
	*J*_ms_	*J*_sm_	*J*_net_	*J*_ms_	*J*_sm_	*J*_net_		
0 (pH 7.4)	26.16 ± 1.75^a,b^ (12)	26.58 ± 2.98^a^ (12)	−0.42 ± 2.96^a^ (12)	16.51 ± 1.06 (8)	14.85 ± 0.95^a^ (8)	1.66 ± 1.38 (8)	−0.29 ± 0.12^a^ (24)	10.45 ± 0.74^a^ (24)
7 (pH 6.9)	27.54 ± 2.72^a^ (11)	37.43 ± 1.81^b^ (11)	−9.89 ± 3.25^a,b^ (11)	15.58 ± 0.75 (8)	11.85 ± 0.32^b^ (8)	3.74 ± 0.80 (8)	−0.53 ± 0.11^a,b^ (20)	11.52 ± 0.77^a,b^ (20)
21 (pH 7.4)	20.75 ± 1.27^b^ (24)	36.70 ± 1.55^b^ (24)	−15.95 ± 1.56^b^ (24)	15.80 ± 0.58 (20)	11.84 ± 0.35^b^ (20)	3.96 ± 0.69 (20)	−0.67 ± 0.07^b^ (54)	13.55 ± 0.63^b^ (54)
42 (pH 7.9)	17.51 ± 2.57^b^ (6)	20.33 ± 1.71^a^ (6)	−2.81 ± 1.94^a^ (6)	13.94 ± 1.28 (6)	12.57 ± 0.87^a,b^ (6)	1.37 ± 1.86 (6)	−1.18 ± 0.15^c^ (12)	11.99 ± 0.72^a,b^ (12)

### Effects of changing pH

The absence of 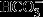 and CO_2_ permitted buffer pH to be manipulated independently of these two variables. Under these circumstances pH did not exert any significant impacts on net oxalate or Cl^−^ transport for either segment examined. For the distal ileum, altering pH between 6.9 and 7.9 did produce a significant increase in I_sc_ from −1.70 to −4.22 *μ*eq/cm^2^/h, which was approximately equivalent in magnitude to the resulting net Cl^−^ secretion at pH 7.9 (5.38 ± 1.67 *μ*mol/cm^2^/h). In addition, this higher pH also produced an increase in G_t_ (Table4). Even though the absence of 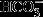 and CO_2_ abolished net oxalate secretion by the distal colon, changing pH under these circumstances did not produce any other dramatic effects on oxalate fluxes by this segment, although 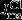 was significantly lower at pH 7.9 this did not translate to a significant change in net Cl^−^ flux (Table5).

**Table 4:** Effects of pH on oxalate and chloride transport by the distal ileum. A comparison of transepithelial oxalate (*J*^Ox^) and Cl^−^ (*J*^Cl^) fluxes measured simultaneously during period I (0–45 min) in 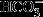/CO_2_-free buffers of different pH across pairs of isolated, short-circuited segments of the distal ileum from wild-type mice. Short-circuit current (*I*_sc_) and transepithelial conductance (*G*_t_) are also shown. Data are mean ± SE and values labeled with a different letter indicate a statistically significant difference. Numbers in parentheses denote associated sample size.

pH	*J*^Ox^ (pmol/cm^2^/h)			*J*^Cl^ (*μ*mol/cm^2^/h)			*I*_sc_ (*μ*eq/cm^2^/h)	*G*_t_ (mS/cm^2^)
	*J*_ms_	*J*_sm_	*J*_net_	*J*_ms_	*J*_sm_	*J*_net_		
6.9	16.77 ± 1.80 (6)	50.71 ± 7.12 (6)	−33.94 ± 7.70 (6)	7.79 ± 0.37 (6)	9.08 ± 1.45 (6)	−1.28 ± 1.75 (6)	−1.70 ± 0.55^a^ (12)	23.91 ± 1.33^a^ (12)
7.4	16.70 ± 2.17 (8)	52.39 ± 5.50 (8)	−35.69 ± 6.78 (8)	7.80 ± 0.65 (8)	10.28 ± 1.15 (8)	−2.49 ± 1.24 (8)	−1.95 ± 0.26^a^ (16)	25.84 ± 1.81^a^ (16)
7.9	21.98 ± 2.76 (5)	51.07 ± 9.55 (5)	−29.09 ± 9.43 (5)	8.17 ± 0.65 (5)	13.55 ± 1.74 (5)	−5.38 ± 1.67 (5)	−4.22 ± 0.52^b^ (10)	32.55 ± 2.28^b^ (10)

**Table 5:** Effects of pH on oxalate and chloride transport by the distal colon. A comparison of transepithelial oxalate (*J*^Ox^) and Cl^−^ (*J*^Cl^) fluxes measured simultaneously during period I (0–45 min) in 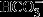/CO_2_-free buffers of different pH across pairs of isolated, short-circuited segments of the distal colon from wild-type mice. Short-circuit current (*I*_sc_) and transepithelial conductance (*G*_t_) are also shown. Data are mean ± SE and values labeled with a different letter indicate a statistically significant difference. Numbers in parentheses denote associated sample size.

pH	*J*^Ox^ (pmol/cm^2^/h)			*J*^Cl^ (*μ*mol/cm^2^/h)			*I*_sc_ (*μ*eq/cm^2^/h)	*G*_t_ (mS/cm^2^)
	*J*_ms_	*J*_sm_	*J*_net_	*J*_ms_	*J*_sm_	*J*_net_		
6.9	27.88 ± 3.77 (6)	30.27 ± 4.47 (6)	−2.38 ± 4.60 (6)	15.69 ± 0.80^a,b^ (6)	14.10 ± 0.61 (6)	1.59 ± 1.08 (6)	−0.66 ± 0.25 (12)	12.09 ± 1.03 (12)
7.4	26.16 ± 1.75 (12)	26.58 ± 2.98 (12)	−0.42 ± 2.96 (12)	16.51 ± 1.06^a^ (8)	14.85 ± 0.95 (8)	1.66 ± 1.38 (8)	−0.29 ± 0.12 (24)	10.45 ± 0.74 (24)
7.9	20.25 ± 3.18 (8)	22.75 ± 2.58 (8)	−2.26 ± 4.07 (8)	12.23 ± 1.06^b^ (8)	12.79 ± 0.73 (8)	−0.55 ± 1.55 (8)	−0.69 ± 0.21 (16)	12.61 ± 0.91 (16)

### Effect of increasing Pco_2_

**Figure 1:**
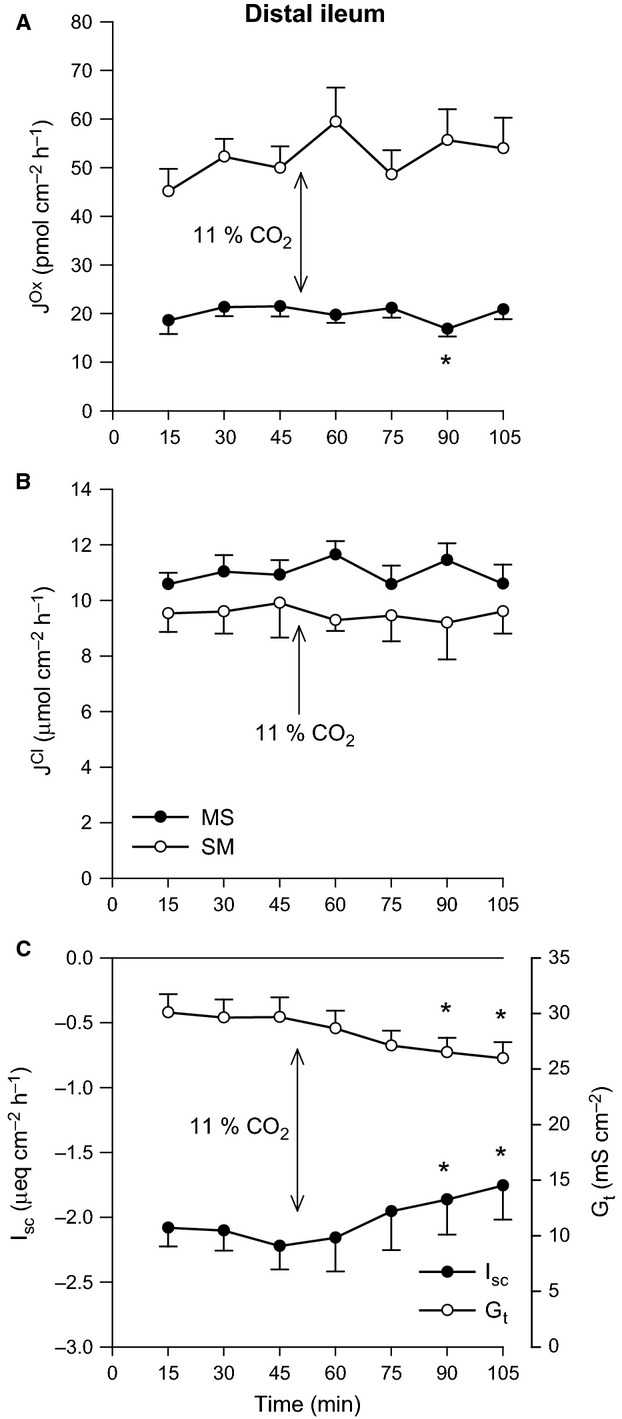
Effects of elevated Pco_2_ on oxalate and chloride transport by the distal ileum. The unidirectional oxalate fluxes, *J*^O^^x^ (pmol/cm^2^/h), and Cl^−^ fluxes, *J*^C^^l^ (*μ*mol/cm^2^/h), measured across isolated, short-circuited segments of distal ileum in standard bicarbonate buffer following an increase in Pco_2_ using 11% CO_2_ (mucosal + serosal), are shown in Panels A and B, respectively. Panel C displays the responses of short-circuit current (*I*_sc_) and transepithelial conductance (*G*_t_). Each data point represents mean ± SE of tissue pairs from *n* = 8 wild-type mice. An asterisk represents a statistically significant change from the preceding control period (0–45 min).

**Figure 2:**
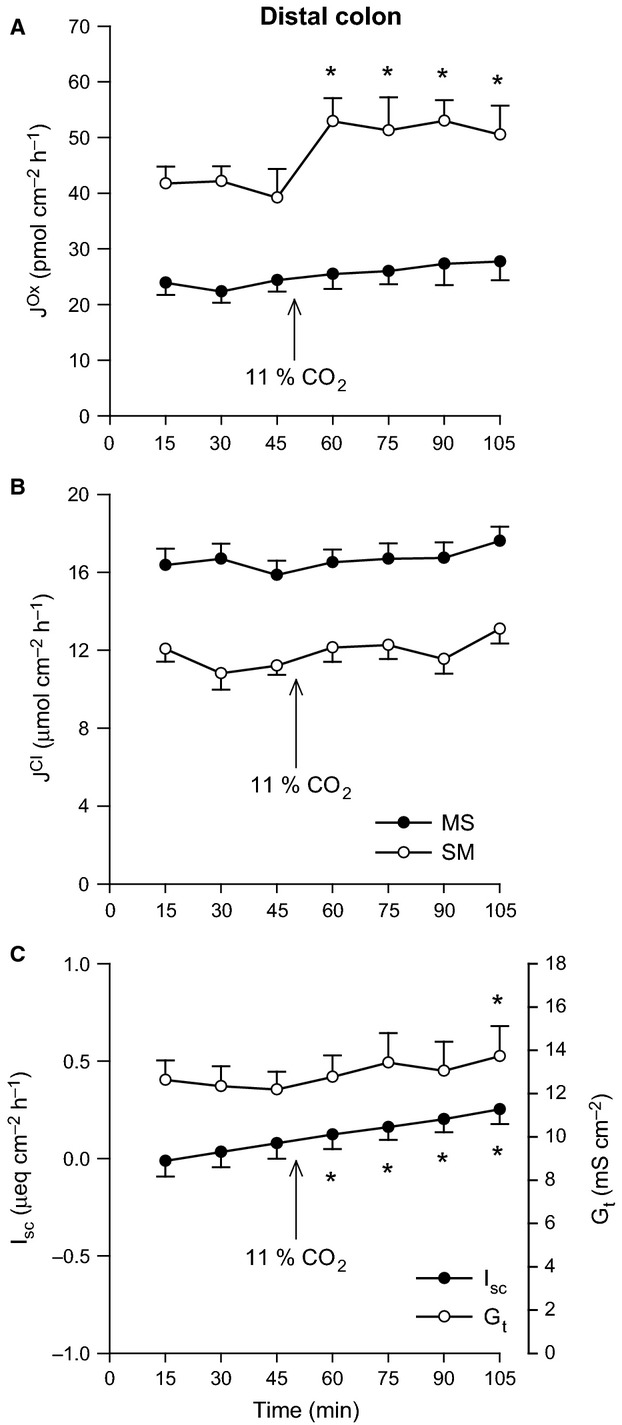
Effects of elevated Pco_2_ on oxalate and chloride transport by the distal colon. The unidirectional oxalate fluxes, *J*^O^^x^ (pmol/cm^2^/h), and Cl^−^ fluxes, *J*^C^^l^ (*μ*mol/cm^2^/h), measured across isolated, short-circuited segments of distal colon in standard bicarbonate buffer following an increase in Pco_2_ using 11% CO_2_ (mucosal + serosal), are shown in Panels A and B, respectively. Panel C displays the responses of short-circuit current (*I*_sc_) and transepithelial conductance (*G*_t_). Each data point represents mean ± SE of tissue pairs from *n* = 8 wild-type mice. An asterisk represents a statistically significant change from the preceding control period (0–45 min).

There were no significant effects of increasing Pco_2_ on oxalate or Cl^−^ fluxes across the distal ileum aside from a reduction in G_t_ (Fig.1). In contrast, 11% CO_2_ produced a clear, rapid increase in the secretory flux of oxalate by the distal colon (Fig.2A) which translated to a significant 41% enhancement of net oxalate secretion from −17.50 ± 3.26 to −24.60 ± 3.13 pmol/cm^2^/h. There were, however, no accompanying changes to Cl^−^ fluxes (Fig.2B), although I_sc_ gradually became positive (Fig.2C).

### Role of carbonic anhydrase

**Figure 3:**
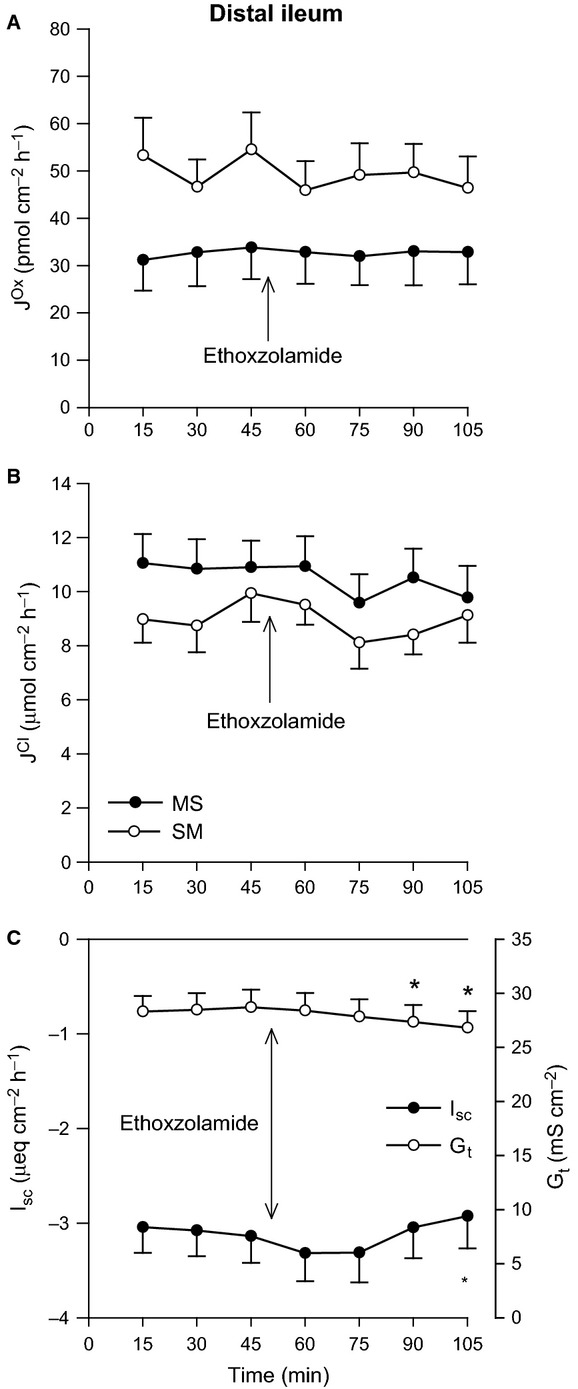
Effects of carbonic anhydrase inhibition on oxalate and chloride transport by the distal ileum. The unidirectional oxalate fluxes, *J*^O^^x^ (pmol/cm^2^/h), and Cl^−^ fluxes, *J*^C^^l^ (*μ*mol/cm^2^/h), measured across isolated, short-circuited segments of distal ileum in standard bicarbonate buffer following application of the carbonic anhydrase inhibitor ethoxzolamide (100 *μ*mol/L, mucosal + serosal), are shown in Panels A and B, respectively. Panel C displays the responses of short-circuit current (*I*_sc_) and transepithelial conductance (*G*_t_). Each data point represents mean ± SE of tissue pairs from *n* = 15 wild-type mice. An asterisk represents a statistically significant change from the preceding control period (0–45 min).

**Figure 4:**
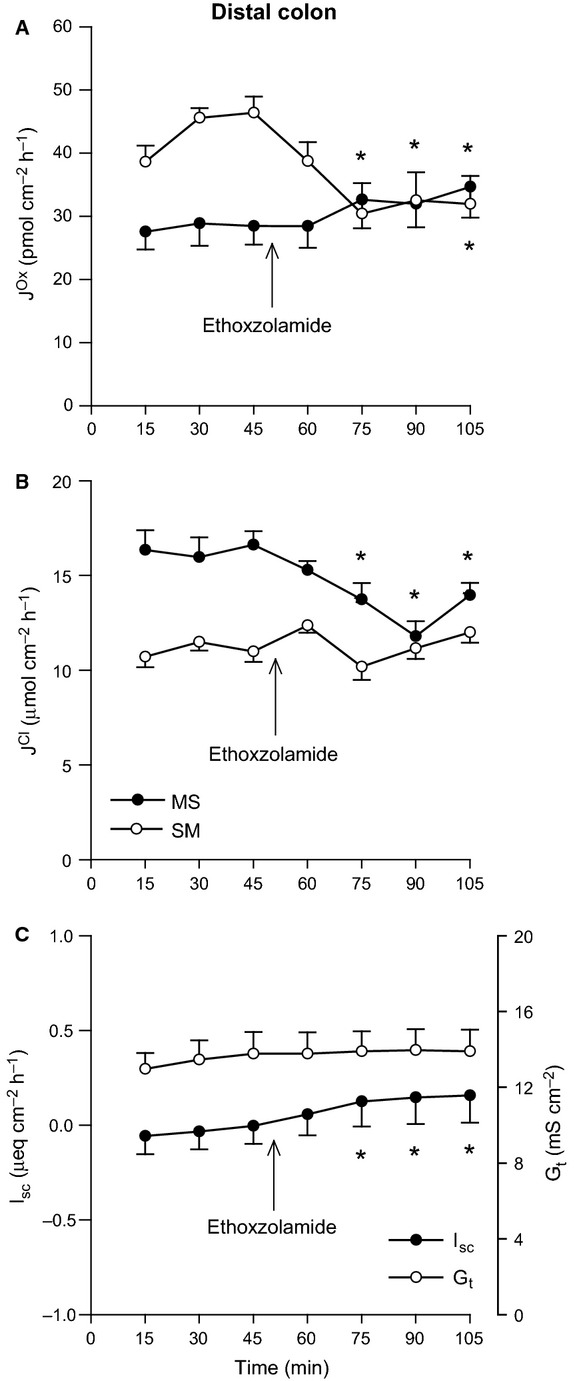
Effects of carbonic anhydrase inhibition on oxalate and chloride transport by the distal colon. The unidirectional oxalate fluxes, *J*^O^^x^ (pmol/cm^2^/h), and Cl^−^ fluxes, *J*^C^^l^ (*μ*mol/cm^2^/h), measured across isolated, short-circuited segments of distal colon in standard bicarbonate buffer following application of the carbonic anhydrase inhibitor ethoxzolamide (100 *μ*mol/L, mucosal + serosal), are shown in Panels A and B, respectively. Panel C displays the responses of short-circuit current (I_sc_) and transepithelial conductance (*G*_t_). Each data point represents mean ± SE of tissue pairs from *n* = 8 wild-type mice. An asterisk represents a statistically significant change from the preceding control period (0–45 min).

**Figure 5:**
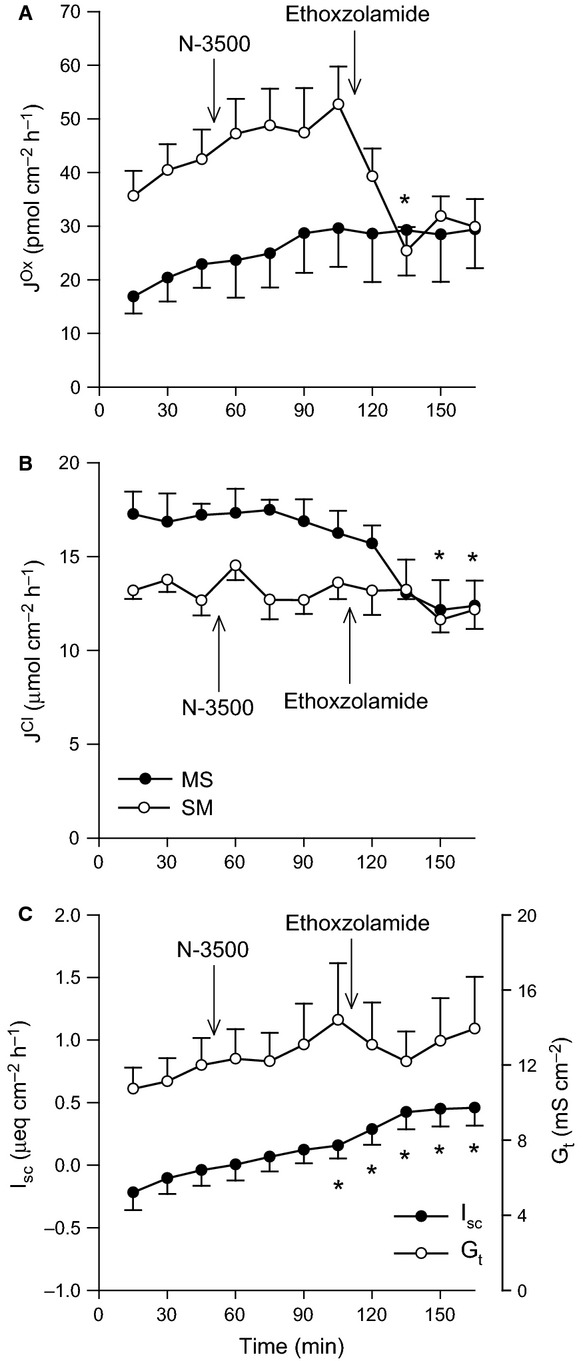
Effects of carbonic anhydrase inhibition on oxalate and chloride transport by the distal colon. The unidirectional oxalate fluxes, *J*^O^^x^ (pmol/cm^2^/h), and Cl^−^ fluxes, *J*^C^^l^ (*μ*mol/cm^2^/h), measured across isolated, short-circuited segments of distal colon in standard bicarbonate buffer following application of the membrane-impermeant carbonic anhydrase inhibitor N-3500 (100 *μ*mol/L, mucosal only), followed by ethoxzolamide (100 *μ*mol/L, mucosal + serosal), are shown in Panels A and B, respectively. Panel C displays the responses of short-circuit current (*I*_sc_) and transepithelial conductance (*G*_t_). Each data point represents mean ± SE of tissue pairs from *n* = 6 wild-type mice. An asterisk represents a statistically significant change from the preceding control period (0–45 min).

In Figure3, application of the CA inhibitor ethoxzolamide did not impact any parameter in the distal ileum, with the exception of G_t_ which showed a very modest reduction (Fig.3C). For the distal colon, there were dramatic changes to oxalate fluxes in response to CA inhibition, where net secretion was completely abolished (Fig.4A). This was primarily through a 27% reduction in 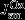, with a smaller rise in 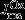. There was also an exclusive decrease in 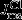, lowering net Cl^−^ absorption by 60% (Fig.4B), accompanied by a modest, but significant change in direction of I_sc_ (Fig.4C). Targeting the external CA at the apical membrane of the distal colon with N-3500 did not significantly diminish 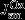, only the subsequent addition of ethoxzolamide was able to inhibit net oxalate secretion (Fig.5A). Similarly, this final maneuver also abolished net Cl^−^ absorption via 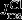, where N-3500 had previously no effect (Fig.5B), and was accompanied by an increasingly positive *I*_sc_ (Fig.5C).

**Figure 6:**
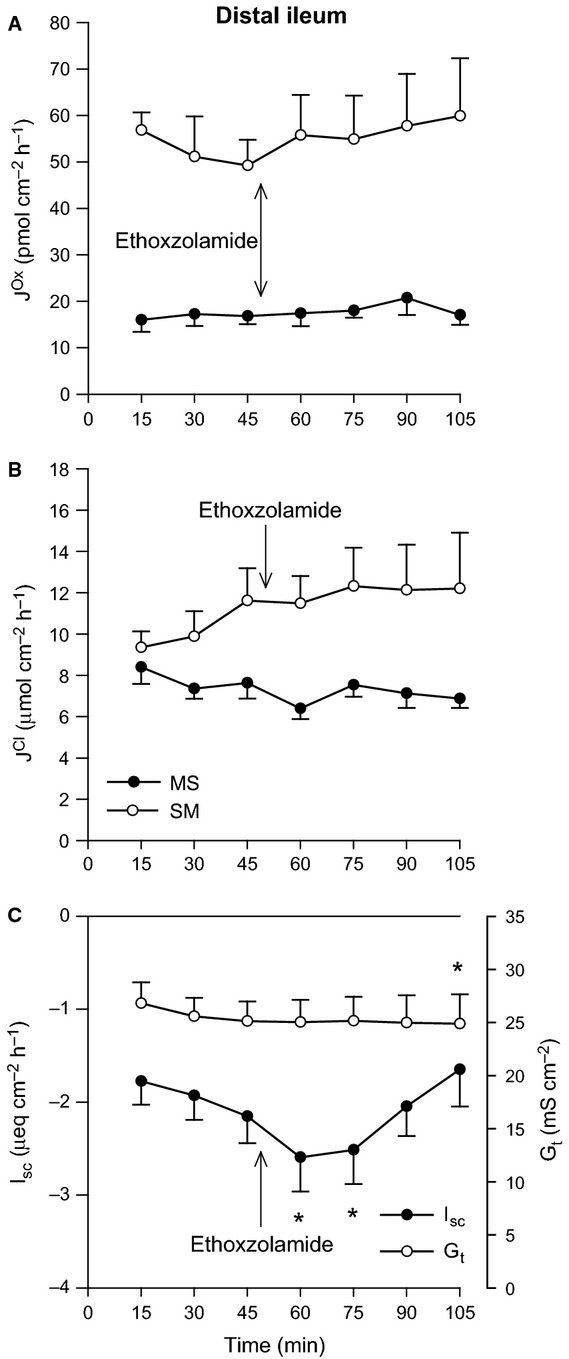
Effects of carbonic anhydrase inhibition on oxalate and chloride fluxes by the distal ileum in HEPES buffer. The unidirectional oxalate fluxes, *J*^O^^x^ (pmol/cm^2^/h), and Cl^−^ fluxes, *J*^C^^l^ (*μ*mol/cm^2^/h), measured across isolated, short-circuited segments of distal ileum in 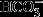/CO_2_-free HEPES buffer following application of the carbonic anhydrase inhibitor ethoxzolamide (100 *μ*mol/L, mucosal + serosal), are shown in Panels A and B, respectively. Panel C displays the responses of short-circuit current (*I*_sc_) and transepithelial conductance (*G*_t_). Each data point represents mean ± SE of tissue pairs from *n* = 8 wild-type mice. An asterisk represents a statistically significant change from the preceding control period (0–45 min).

**Figure 7:**
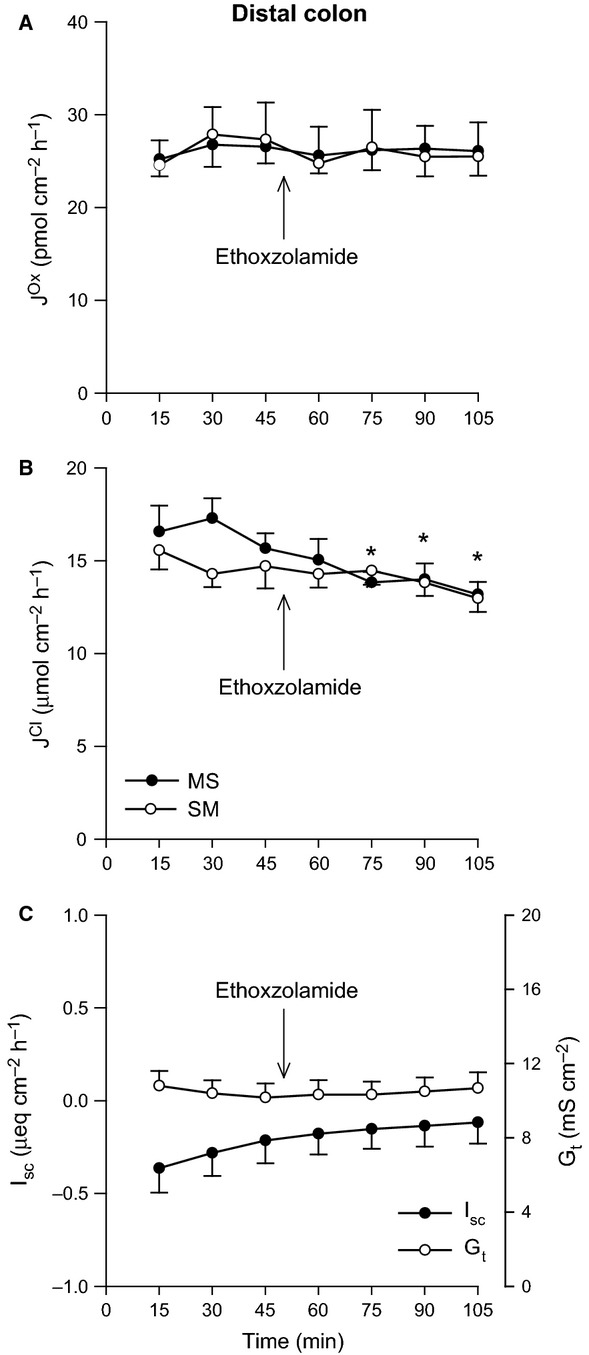
Effects of carbonic anhydrase inhibition on oxalate and chloride fluxes by the distal colon in HEPES buffer. The unidirectional oxalate fluxes, *J*^O^^x^ (pmol/cm^2^/h), and Cl^−^ fluxes, *J*^C^^l^ (*μ*mol/cm^2^/h), measured across isolated, short-circuited segments of distal colon in 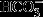/CO_2_-free HEPES buffer following application of the carbonic anhydrase inhibitor ethoxzolamide (100 *μ*mol/L, mucosal + serosal), are shown in Panels A and B, respectively. Panel C displays the responses of short-circuit current (*I*_sc_) and transepithelial conductance (*G*_t_). Each data point represents mean ± SE of tissue pairs from *n* = 12 wild-type mice. An asterisk represents a statistically significant change from the preceding control period (0–45 min).

When ethoxzolamide was used to inhibit CA in the absence of extracellular 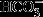/CO_2_, the distal ileum proved itself refractory to this maneuver also, with no significant effects on oxalate or Cl^−^ transport (Fig.6A and B), although there was a transient increase in I_sc_ (Fig.6C). The absence of net oxalate secretion by the distal colon under 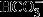/CO_2_-free conditions did not change following the application of ethoxzolamide with no subsequent effect on unidirectional fluxes (Fig.7A). The reduction in 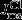 seen previously with CA inhibition in standard 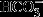 buffer was also evident in HEPES buffer and eliminated net Cl^−^ absorption (Fig.7B).

## Discussion

Previous work comprehensively established that states of acute respiratory and metabolic acidosis or alkalosis produce rapid, reversible alterations to NaCl absorption, 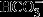 secretion and fluid handling by the ileum and colon in vivo. These effects were subsequently shown to be specific responses by the apical Na^+^/H^+^ and Cl^−^/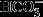 exchangers to extracellular 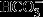 concentration ([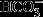]_e_), Pco_2_ and pH, through corresponding alterations to intracellular pH (pH_i_) and 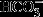 ([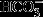]_i_), mediated by CA. As oxalate is a substrate of the intestinal Cl^−^/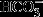 exchangers, the aim of this study was to systematically examine how these acid-base variables impacted oxalate transport by the mouse intestine in vitro. Under standard buffer conditions we found the distal ileum and distal colon each mediated net oxalate secretion and Cl^−^ absorption, but only the latter segment and notably the 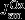 pathway, were exclusively responsive to changes in 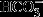
_e_, Pco_2_ and dependent on CA activity. Oxalate secretion, but not Cl^−^ absorption, by the distal colon was acutely stimulated by increasing Pco_2_. These findings highlight the distinct segmental heterogeneity of oxalate transport in the intestine, provide important new insights into the characteristics of the transport mechanism, and strongly imply that oxalate secretion by the mouse distal colon is specifically regulated by CO_2_.

### Effects of acid-base variables on Cl^−^ transport by the mouse intestine

The responses of intestinal Na^+^ and Cl^−^ transport to pH, 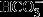 and CO_2_, and the role of CA, have been studied extensively in the rat model, and to a lesser degree in the mouse. As this was the first study investigating the relationship between oxalate and these acid-base variables in the mouse, simultaneously measuring Cl^−^ fluxes provided important points of reference to these earlier investigations and assisted subsequent interpretation of the associated oxalate fluxes. Net Cl^−^ absorption by the rat distal ileum in vivo, and specifically 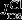 in vitro, was indirectly proportional to extracellular pH, whether produced by alterations in Pco_2_, [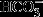]_e_ or in 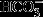/CO_2_-free HEPES buffer (Charney and Feldman 1984; Kurtin and Charney 1984; Wagner et al. 1986; Vaccarezza and Charney 1988; Charney et al. 1991), and also dependent on CA (Charney et al. 1986, 2002a). Subsequent work on this segment in the mouse showed that increasing Pco_2_ from 21 to 70 mmHg stimulated net Cl^−^ absorption in identical fashion (Charney et al. 2004b). In contrast, we found ileal Cl^−^ fluxes were unresponsive when subjected to a similar elevation of Pco_2_ (Fig.1B). Reducing pH from 7.61 to 7.09 in HEPES buffer stimulated 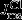 by the rat ileum 16% (Vaccarezza and Charney 1988), yet we found Cl^−^ fluxes by the mouse ileum were not significantly altered between pH 6.9 and 7.9 (Table4). The absence of 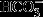/CO_2_ reversed net Cl^−^ absorption to net secretion exclusively through a reduction in 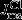 (Table2), indicating endogenous 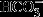 production was insufficient to support apical Cl^−^/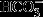 exchange and was thus dependent on 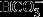 supplied from the serosal bath. This notion was corroborated by the inability of CA inhibitor ethoxzolamide to impact Cl^−^ fluxes (Figs.3B and 5B), and consistent with the abolition of DIDS-sensitive (mucosal Cl^−^-dependent) 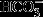 secretion by the mouse distal ileum following the removal of serosal 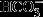/CO_2_ (Uchiyama et al. 2007; Zhang et al. 2007). A role for CA in the mouse ileum cannot be dismissed entirely as Uchiyama et al. (2007) observed that Cl^−^-dependent 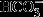 secretion was reduced ∼ 30% in the presence of 100 *μ*mol/L acetazolamide. In contrast, net Cl^−^ absorption by the rat ileum was sustained in the absence of 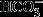/CO_2_ (Vaccarezza and Charney 1988), and furthermore was sensitive to methazolamide (Charney et al. 2002a), indicating a prominent contribution of CA to ileal transport.

These acid-base variables also exert their effects on Na^+^ and Cl^−^ absorption by the rat distal colon in vitro in a similar manner (Goldfarb et al. 1988), a segment characterized as sensitive to CO_2_ rather than pH (Charney and Dagher 1996; Charney et al. 2004a). Subsequent work on the mouse distal colon in vitro revealed trends largely consistent with the rat, where raising Pco_2_ to 69 mmHg exclusively stimulated 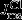 (Goldfarb et al. 2000; Charney et al. 2004b), while reducing pH from 7.61 to 7.09 (in HEPES buffer) notably enhanced net Cl^−^ absorption through a reduction in 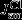 (Goldfarb et al. 2000). Unlike these previous studies, we found increasing Pco_2_ did not stimulate Cl^−^ fluxes by the distal colon (Fig.2B), and net Cl^−^ absorption was also independent of pH (Table4). In the absence of 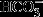/CO_2_ (pH 7.6), net Cl^−^ absorption in the rat and mouse distal colon was abolished due to a reduction of 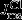, relative to standard 21 mmol/L 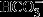 buffer (Goldfarb et al. 1988, 2000; Charney et al. 2004a), indicating a limited contribution from metabolic 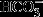 production. Conversely, Table2 shows 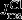 and 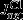, were independent of 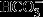/CO_2_, although interestingly the secretory 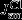 flux was significantly higher in this HEPES buffer (discussed further on). In our study, 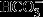
_i_ was therefore sufficient to maintain apical Cl^−^/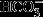 exchange (represented by 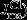, in the absence of external 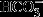/CO_2_, thus implying a substantial contribution from CA. This is supported by the observation that on average, ethoxzolamide reduced 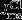 by a similar magnitude (∼ 3–4 *μ*mol/cm^2^/h) whether 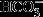/CO_2_ was present (Fig.4B and 5B) or not (Fig.6B). However, if CA is providing 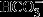
_i_ from CO_2_ to drive apical Cl^−^/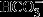 exchange, then it is curious as to why increasing Pco_2_ failed to stimulate 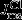 (Fig.2B) as shown reproducibly in previous studies, particularly as [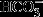]_i_ increases from 11.3 to 18.3 mmol/L when Pco_2_ is raised from 21 to 69 mmHg (Dagher et al. 1992).

The reasons for the above discrepancies between our present work on the mouse and previous results are not clear. In relation to studies with rats, species-specific differences in the functional expression of the Cl^−^ transporter(s), particularly the anion exchanger, AE1 (Slc4a1), may be one explanation. Both DRA and AE1 are present in the rat colon (Rajendran et al. 2000), where they are suggested to operate as respective Cl^−^/OH^−^ and Cl^−^/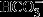 exchangers at the apical membrane, thus endowing AE1 as the principal Cl^−^ transporter (Rajendran and Binder 1999, 2000). This is significant considering the CO_2_-stimulated increase in 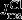 by the rat distal colon correlated with enhanced AE1 expression (Charney et al. 2004a). In contrast to a prominent role for AE1 in the rat large intestine, there is no evidence that this is the case for the mouse. Utilizing immunocytochemistry, Alper et al. (1999) found that antibodies directed against AE1 did not stain enterocytes from the mouse colon, and thus lacks AE1 expression. Although we note that more recent work has detected AE1 mRNA in the mouse large intestine (Gawenis et al. 2010). If AE1 is absent from the mouse intestine this might explain why increasing CO_2_ failed to stimulate Cl^−^ absorption in this study. For the mouse (and human) intestine, DRA rather than AE1 has become recognized as the main Cl^−^/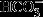 exchanger (Hoglund et al. 1996; Schweinfest et al. 2006; Kato and Romero 2011; Freel et al. 2013). Qualitatively, the mouse and rat share a near-identical pattern of DRA expression along the large intestine (Talbot and Lytle 2010), yet the relative contributions of DRA and AE1 to Cl^−^ transport for the latter model remain undetermined. In addition to species-specific differences, our data also showed similar departures from other mouse studies (Goldfarb et al. 2000; Charney et al. 2004b). As strain-related differences in intestinal transport do exist between mice this might offer some explanation. For example, duodenal calcium and phosphate absorption by C57BL/6 mice were found to be different from C3H/He mice (Armbrecht et al. 2002), whereas the stimulation of colonic ion secretion in Sv129 mice was distinct from the C57BL strain (Flores et al. 2010). For both of these studies the disparities between strains were, in part, the result of differences in respective transporter expression. A similar scenario, perhaps related to functional AE1 expression, may explain why Cl^−^ absorption by the ileum and colon of the Balb/C mouse used by Charney et al. (2004b) responded to Pco_2_, but the C57BL/6 strain used here did not. We note that AE1 expression was absent from the large intestine of CD1 mice (Alper et al. 1999), whereas AE1 mRNA was detected in the colon of mice on a mixed 129SvEv/Black Swiss background (Gawenis et al. 2010). However, this line of reasoning becomes somewhat uncertain in relation to the findings of Goldfarb et al. (2000), who presented data pooled from C57BL/6J and DBA/2J mice, based on no measurable differences in Na^+^ and Cl^−^ flux rates, CA isozyme expression or CA activity between strains.

### Oxalate secretion by the distal colon is stimulated by CO_2_

Although elevated Pco_2_ unexpectedly failed to stimulate 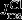, it dramatically enhanced oxalate secretion (Fig.2A). A major portion (70–75%) of CO_2_-stimulated NaCl absorption by the rat distal colon corresponds to the CA-dependent trafficking of NHE3 and the anion exchanger AE1 (Slc4a1) to the apical membrane (Charney et al. 2002b, 2004a, 2002b). As 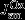 was also acutely stimulated by CO_2_ (Fig.2A), and dependent on CA (Fig.4A), independent of any changes in G_t_ (Figs.2C and 4C), we considered whether alterations to membrane transporter expression might also explain this response. If this increase in 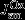 was due to changes in an apical Cl^−^/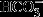 exchanger such as AE1 or DRA, then we would anticipate an accompanying increase in 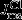, but this was not the case (Fig.2B). Furthermore, while DRA accounts for 50% of 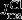 in the mouse distal colon, we have shown that it is involved in transcellular oxalate absorption rather than secretion (Freel et al. 2013), and notably Pco_2_ was also without effect on 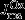 (Fig.2A). While PAT1 has been identified as the apical anion exchanger responsible for oxalate secretion by the small intestine (Freel et al. 2006; Jiang et al. 2006), the apical transporter(s) involved in the large intestine have not been resolved. Considered to be most prominent in the small intestine, PAT1 expression does extend into the large intestine (Wang et al. 2002; Hatch et al. 2011), yet its function there is uncertain. We have recently shown PAT1 contributes to sulfate (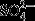) secretion by the mouse cecum (Whittamore et al. 2013), but whether this also applies to oxalate and the distal colon has yet to be revealed. Interestingly, PAT1 is considered responsive to systemic acid-base status, as PAT1-mediated 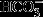 secretion by the mouse duodenum in vivo was decreased when the systemic acidosis induced by isoflurane anesthesia was left uncorrected (Singh et al. 2008). Although PAT1 contributes to 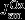 in the distal ileum (Freel et al. 2006), we have shown here that oxalate secretion by this same segment was unaffected when subjected to acidotic conditions in vitro, that is, where [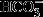] = 7 mmol/L and pH 6.9 (Table3), and following an increase in Pco_2_ (Fig.1A). Previous work on the rabbit distal colon showed that 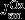 and net oxalate secretion could be stimulated by cAMP with characteristics bearing resemblance to electrogenic Cl^−^ secretion (Hatch et al. 1994). Notably, CO_2_ can elicit cAMP production by recombinant mammalian transmembrane adenylyl cyclases (tmACs) with an EC_50_ of ∼2 mmol/L (Townsend et al. 2009), which is similar to the [CO_2_] achieved with 11% CO_2_ here (2.1 mmol/L). In contrast, CO_2_-stimulated oxalate secretion by the mouse distal colon appeared to be independent of a cAMP-mediated pathway, as Cl^−^ fluxes (Fig.4B) and I_sc_ (Fig.4C) indicated no substantial induction of Cl^−^ or 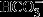 secretion. Furthermore, the application of 10 *μ*mol/L forskolin (a potent agonist of the tmACs) does not stimulate oxalate secretion by the mouse distal colon (Whittamore, J. M. and Hatch, M., unpublished observations).

### Intracellular bicarbonate mediates changes to intestinal ion transport

A common factor linking previous studies on the regulation of intestinal Cl^−^ transport to our present observations on oxalate, and to some extent Cl^−^ fluxes, is [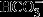]_i_. We found 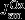 was exclusively sensitive to changes in [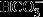]_e_ and Pco_2_, maneuvers that each result in proportional changes to [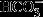]_i_. Every 1 mmol/L increase in [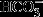]_e_ at a constant Pco_2_ (32 mmHg) has been determined to produce a corresponding 0.54 mmol/L increase in [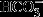]_i_, while every 1 mmHg rise in Pco_2_ increases [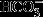]_i_ by 0.12 mmol/L (Dagher et al. 1992). In the rat distal colon [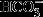]_i_ modulates basal and carbachol-stimulated Cl^−^ secretion (Dagher et al. 1992, 1994), where 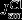 was inversely related to [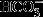]_i_ above or below a physiological “plateau” of 9–18 mmol/L (Dagher et al. 1992). We too observed that 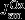 conferred net oxalate secretion between 7 and 21 mmol/L 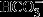 (Table3), and 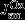 could be stimulated by increasing Pco_2_ (Fig.2A), corresponding to an estimated [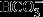]_i_ within a very similar range of 8–20 mmol/L. Either side of this range 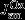 was reduced and net oxalate secretion abolished (Table3). Lowering [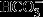]_e_ from 21 mmol/L to zero at pH 7.4 increased 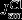 by the rat distal colon 65%, from 7.9 to 13.0 *μ*mol/cm^2^/h (Dagher et al. 1992). We too found a similar effect in HEPES buffer where 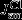 significantly increased ∼ 25% from 11.85 to 14.85 *μ*mol/cm^2^/h (Table3). In addition to 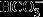, intracellular CA activity was also required for 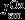 (Figs.4A and 5A), a trait shared with the rat distal colon where both [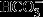]_i_ and CA are crucial mediators for CO_2_-stimulated Cl^−^ absorption (Charney and Dagher 1996) culminating in the trafficking of AE1 to the apical membrane (Charney et al. 2004a). The underlying signaling mechanism(s) regulating intestinal Cl^−^ absorption and secretion by the rat colon have not been determined, and while we have yet to identify the transporter(s) responsible for oxalate secretion by the mouse large intestine, it is interesting to consider whether this secretion might share the same regulatory pathway(s) involving [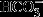]_i_ and CA.

### Possible transport mechanisms involved in intestinal oxalate transport

**Figure 8:**
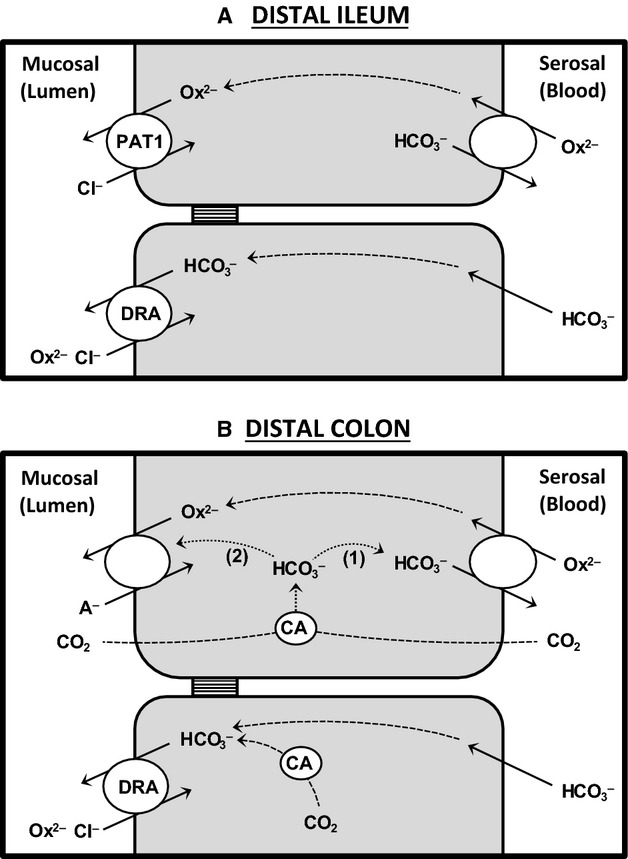
A simple model summarizing some of the known and proposed transcellular pathways for oxalate transport across the distal ileum and distal colon of the mouse intestine. In panel A, oxalate secretion by the ileum was unaffected by the absence of extracellular 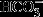/CO_2_ and did not require carbonic anhydrase (CA), but does involve Cl^−^/Ox^2−^ exchange by PAT1 (Slc26a6), which may be supplied by a basolateral 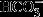/Ox^2−^ exchanger. DRA (Slc26a3) contributes to Cl^−^ (and oxalate) absorption by the ileum driven by the supply of serosal 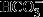, and does not depend on CA activity. In panel B, oxalate secretion by the distal colon required intracellular CA activity which may have been supplying 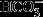 for basolateral 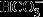/Ox^2−^ exchange. Elevated CO_2_ (via CA and intracellular 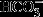) might stimulate oxalate secretion by: (1) promoting basolateral 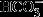/Ox^2−^ exchange, and/or (2) influencing the expression of the apical exchanger responsible for oxalate efflux. DRA contributes to the absorption of Cl^−^ and oxalate by the distal colon, but only Cl^−^ (not oxalate) absorption demonstrated dependence on intracellular CA activity. See text for further details. A^− ^= Cl^−^ or 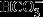.

While [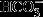]_i_ can modulate Cl^−^ transport, and potentially oxalate secretion, there are additional considerations for interpreting the effects of [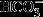]_e_ in relation to anion exchange. As 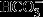 and oxalate are potential substrates on the same transporter, the changes in oxalate fluxes may also be related to direct competition between the two anions (i.e., 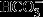 (Ox^2−^)/A^−^ exchange), and/or the dependence of oxalate on 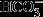 (i.e., 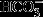/Ox^2−^ exchange), as illustrated in Figure8. In the ileum PAT1 is involved in oxalate secretion, specifically 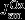, operating as an apical Cl^−^/Ox^2−^ exchanger (Freel et al. 2006). Table2 shows that 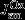 was undiminished by reducing, or removing, 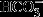/CO_2_ from the buffer thus indicating PAT1 does not also perform 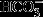/Ox^2−^ exchange in the ileum. Even though PAT1 is capable of a variety of transport modes when expressed in *Xenopus* oocytes, including 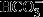/Ox^2−^ exchange (Chernova et al. 2005), rates are considered modest relative to Cl^−^/Ox^2−^ exchange (Clark et al. 2008), consistent with our observations. Interestingly, recent work has suggested PAT1 contributes to 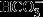 re-absorption by the jejunum via 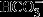
_o_/Cl^−^_i_ exchange (Xia et al. 2014), and could therefore conceivably perform 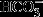/Cl^−^ (Ox^2−^) exchange in this part of the small intestine. Unlike the ileum, 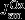 and consequently net oxalate secretion by the distal colon was clearly dependent on the presence of 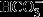/CO_2_ (Table3). One explanation would therefore be if oxalate was dependent on 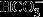
_e_ and exiting across the apical membrane via 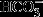
_o_/Ox^2−^_i_ exchange (Fig.8B).

There were also distinct reductions to 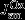 at 42 mmol/L 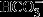, for both the ileum (Table2) and distal colon (Table3), which were independent of the change to pH (Tables4 and 5), and thus considered specific to 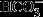. As oxalate secretion at the apical membrane of the distal ileum (via PAT1) is not 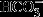-dependent, this may represent an impact on a basolateral anion exchanger. For example, oxalate might be competing with 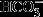 in the serosal bath for entry into the cell by Cl^−^/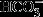 (Ox^2−^) exchange, since at 42 mmol/L 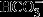, 1.5 *μ*mol/L oxalate would be further out-competed by 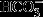. Basolateral membrane vesicles from rabbit ileum identified a distinct 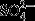/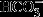 (Ox^2−^) exchanger (Knickelbein and Dobbins 1990), but it is not known whether a similar oxalate transporter is present in the mouse ileum. Presently, very little is known about oxalate transport at the basolateral membrane, but a candidate is SAT1 (Sulfate Anion Transporter 1; Slc26a1). SAT1 has been characterized as a 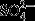/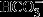 (Ox^2−^) exchanger (Karniski et al. 1998; Krick et al. 2009), expressed in the ileum and proposed to contribute to intestinal 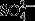 absorption and oxalate secretion (Dawson et al. 2010). Although if a transport mode such as this were operating, one could reasonably argue that in 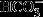/CO_2_ free conditions (with no competition from 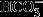), 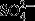/Ox^2−^ exchange might prevail and thus 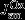 would have been enhanced, or at the very least sustained, but this was not the case for either the ileum (Table2) or colon (Table3). An alternative could be if 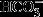
_i_ were the counter-ion for transport across the basolateral membrane (i.e., 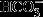
_i_/Ox^2−^_o_ exchange), as illustrated in Figure8 for both the ileum and distal colon. The outwardly directed 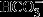 gradient driving this exchange would therefore be greatly diminished with extracellular 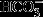 at 42 mmol/L.

### Role of carbonic anhydrase in intestinal oxalate transport

In the intestine, CA can supply intracellular H^+^ and 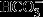 for apical Na^+^/H^+^ and Cl^−^/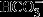 exchange, therefore its contribution to NaCl absorption and 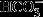 secretion is intuitively recognized. We have now extended this role for CA to include oxalate secretion by the mouse distal colon which was dependent on CA activity, evidenced by the ability of ethoxzolamide to abruptly reduce 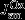 (Figs.4A and 5A). This was only observed in the presence of extracellular 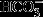/CO_2_, and not in HEPES buffer (Fig.6A), ruling out the possibility of a noncatalytic role for CA in oxalate secretion, which has been suggested for apical Cl^−^/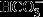 exchange in the rat ileum (Charney et al. 2002a), and mouse distal colon (Goldfarb et al. 2000). Of the 16 mammalian CA isozymes identified, the large intestine prominently expresses three, CAI and II (intracellular) and CAIV, an extracellular form bound to the apical membrane (Lonnerholm et al. 1985; Fleming et al. 1995; Goldfarb et al. 2000). With no isoform-specific, or membrane-impermeant, inhibitors commercially available, distinguishing the relative contributions of each is challenging. Utilizing a CAII-KO mouse model and the relatively impermeant CA inhibitor benzolamide, Goldfarb et al. (2000), concluded CAI was required for optimal NaCl absorption by the distal colon. The failure of mucosal N-3500 to abolish net Cl^−^ absorption, relative to the highly permeant ethoxzolamide (Fig.5B), would further support this notion of an intracellular CA isoform supporting apical Cl^−^/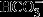 exchange. The CA-dependence of 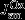 could be interpreted as a function of the extracellular CAIV facilitating apical 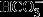/Ox^2−^ exchange. In this scenario the mucosal application of N-3500 might be expected to reduce 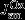, but this was clearly not the case (Fig.5A), subsequently pointing to a role for an intracellular CA isoform. We suggest that the dependence of oxalate secretion on CA, may be through its supply of 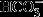
_i_ to a basolateral 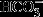/Ox^2−^ exchanger driving oxalate into the cell, and this may also explain the ability of CO_2_ to stimulate oxalate secretion in the distal colon (Fig.8B).

Another possible mechanism supporting a role for CA in oxalate secretion by the distal colon may be the ability of some CA isoforms, including CAII and CAIV, to physically and functionally interact with a number of the oxalate-transporting exchangers, notably PAT1 (Alvarez et al. 2005) and DRA (Sterling et al. 2002). Figures4B and 5B show 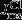 was also simultaneously inhibited by ethoxzolamide and may reflect a reduction in Cl^−^/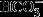 exchange by DRA. However, despite its role in oxalate absorption (Freel et al. 2013) we noted 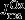 was not subject to similar inhibition (Figs.4A and 5A). The characterization of PAT1 as an ileal Cl^−^/Ox^2−^ exchanger (Freel et al. 2006), and independent of 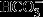/CO_2_ (discussed above), would be consistent with the inability of CA to impact 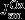 in the distal ileum (Fig.3A), despite the fact that PAT1 can physically bind to CAII (Alvarez et al. 2005). Furthermore, AE1 is another Cl^−^/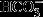 exchanger capable of physically, as well as functionally, associating with CAII (Sowah and Casey 2011), and involved in CO_2_-stimulated Cl^−^ absorption by the rat distal colon (Charney et al. 2004a), where it is expressed with, and functions alongside, DRA (Rajendran and Binder 2000). In human erythrocytes AE1 can exchange oxalate for Cl^−^ (Jennings and Adame 1996), but its contribution as either an oxalate or Cl^−^ transporter in the mouse intestine has not been specifically examined, and there is doubt about whether it is even expressed in the colon (Alper et al. 1999).

### Perspectives and summary

Systemic acid-base imbalances lead to coordinated adjustments by various organ systems (cardiovascular, renal, and skeletal) to compensate and restore homeostasis. The contribution of the intestine is easily over-looked, but it too can respond, most notably through the Na^+^/H^+^ and Cl^−^/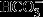 exchangers (Charney and Feldman 1984; Charney and Dagher 1996). This is not surprising given the intestine and its resident transporters are involved in handling significant amounts of acidic and basic equivalents each day, and systemic acid-base disturbances are well-documented complications of many gastrointestinal disorders (Charney et al. 1995; Gennari and Weise 2008). There is very little information on the extent to which acute or chronic acid-base disorders will impact intestinal oxalate transport in vivo, and what consequences (if any) this may have for overall oxalate homeostasis. In genetically hypercalciuric rats, induction of a chronic metabolic acidosis significantly reduced urinary oxalate excretion (Bushinsky et al. 2001). However, similar chronic acid loading of normocalcemic rats did not reveal any significant changes to either urinary oxalate excretion or serum oxalate (Green et al. 2005), suggesting a systemic acidosis does not alter renal oxalate handling or oxalate metabolism. The DRA-KO mouse is a model of the disease congenital chloride diarrhea (CCD) and exhibits a chronic metabolic alkalosis with respiratory compensation (Walker et al. 2008; Xiao et al. 2014). In our report on this model (Freel et al. 2013), urinary oxalate excretion and serum oxalate were decreased, associated with an induction of net oxalate secretion by the intestine. We emphasize that this change in transport was due to a reduction in 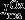 from the absence of DRA, rather than enhanced secretion, but we did record a (nonsignificant) 40% increase in 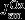 by the distal colon (*P* = 0.10). The intestinal phenotype and urine pH of these mice suggested they harbored the same acid-base disturbance but we did not perform a blood-gas analysis to verify their overall acid-base status. The impact of CCD on the mass-balance of oxalate in humans has not been assessed. A survey of 35 patients diagnosed with, and treated for, this disease found urinary oxalate excretion was within the “normal” range (Wedenoja et al. 2008), however, only very few of these individuals exhibited any systemic acid-base abnormalities (median serum 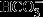
^ ^= 25 mmol/L, blood pH and Pco_2_ were not reported). Finally, CA inhibitors, such as acetazolamide, are used clinically for treating various disorders including glaucoma, edema, seizures, and altitude sickness. A potential complication for patients is the development of metabolic acidosis and a propensity for calcium phosphate kidney stone formation, associated with increased urine pH and hypocitraturia (Matlaga et al. 2003; Mirza et al. 2009). The impacts of CA inhibitors on oxalate homeostasis in vivo, however, are limited and inconclusive. A significant rise in urinary oxalate excretion and some mixed calcium phosphate/oxalate stones have been reported in patients taking acetazolamide (Ahlstrand and Tiselius 1987), whereas other studies with CA-inhibiting drugs have revealed no changes to urinary oxalate handling (Higashihara et al. 1991; Welch et al. 2006; Kaplon et al. 2011).

In summary, oxalate secretion (but not Cl^−^ absorption) by the mouse distal colon was acutely stimulated by increasing Pco_2_ in vitro. The secretory pathway, 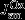, was found to be exclusively responsive to changes in 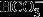, CO_2_, and dependent on CA activity, but not pH. These results strongly suggest oxalate secretion by this segment is specifically regulated by CO_2_. In contrast, net oxalate secretion by the ileum was generally unresponsive to alterations in these same acid-base variables. These findings highlight some of the distinct segmental heterogeneity in oxalate transport that exists along the intestine, but also provides important new insights into the characteristics of the underlying transport mechanisms and how they might be regulated, thus helping to direct future work in this area.
